# Supplementary material for: Lactobacillus fermentum LF31 Supplementation Reversed Atrophy Fibers in a Model of Myopathy Through the Modulation of IL-6, TNF-α, and Hsp60 Levels Enhancing Muscle Regeneration
Source: Nutrients. 2025 Apr 30;17(9):1550. doi: 10.3390/nu17091550 (PMC12073311; doi:10.3390/nu17091550)
Supplement: Supplementary file 1 [file nutrients-17-01550-s001.zip › nutrients-3604101-supplementary.pdf]

nutrients

MDPI

Supplementary Materials: The following supporting information can be downloaded at: https://www.mdpi.com/article/10.3390/nu17091550/s1. Figure S2: The image shows cross-sections of the *red gastrocnemius* muscle stained with Masson's trichrome to assess the fibrosis process. Representative images show muscle fibers with polygonal morphology, characterized by a deep red cytoplasm, indicative of myofiber staining. Connective tissue (in green) is visible in the endomisial and perimysial spaces, separating the muscle fibers. In the two timepoints and the three experimental groups, no particular signs of fibrosis are observed, other than those physiologically present within the muscle tissue. Scale bar 50 µm. Ctrl: control, standard diet; EtOH: 8 or 12 weeks of ethanol in the standard diet; EtOH + Probiotic: 8 or 12 weeks of ethanol plus *L. fermentus* LF31 in the standard diet.

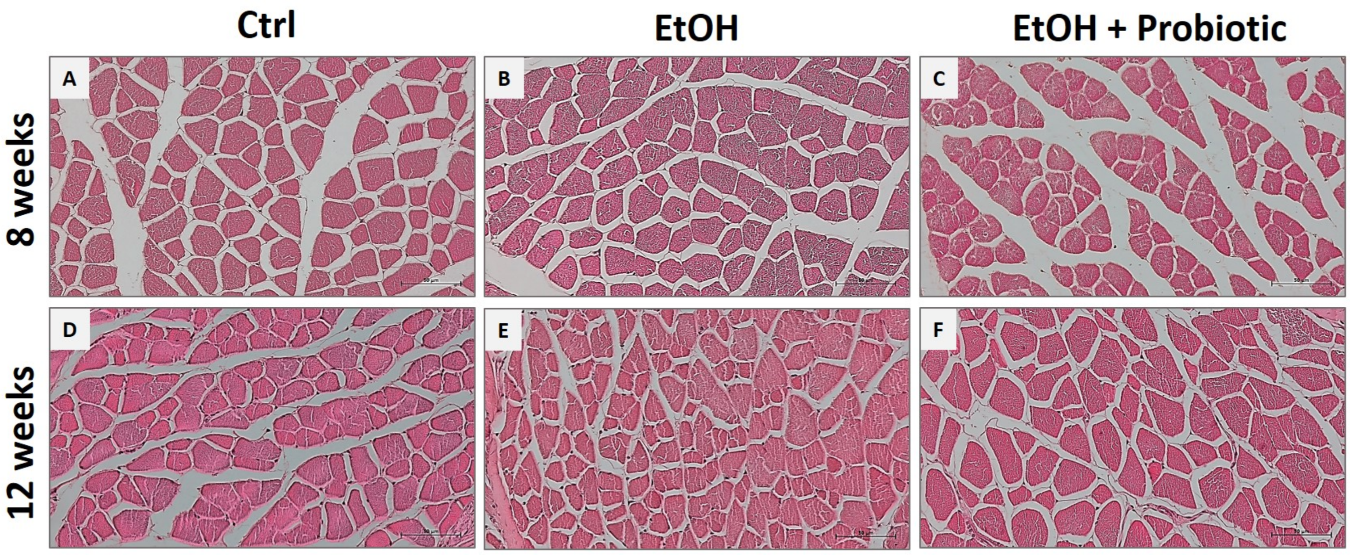

Figure S1. Ematoxilin-eosin stain of cross-section of red *gastrocnemius* muscle. Representative images show cross-sections of the *red gastrocnemius* muscle at two different time points (8 and 12 weeks) and in three experimental groups: Ctrl, EtOH and EtOH+P. Under physiological conditions (A,D), muscle fibers appear regularly organized, with polygonal cross-section, relatively uniform size and peripheral nucleus. In the EtOH group (B,E), a reduction in fiber caliber, greater dimensional variability, more irregular shape and disorganization of muscle architecture are observed, indicative of muscle atrophy and tissue stress. In the EtOH+P group (C,F), muscle fibers show greater uniformity than in the EtOH group, with an increase in fiber caliber and better tissue organization, suggesting precisely a protective and regenerative effect of *L. fermentum* on muscle architecture. Magnifiction 200X. Scale bar 50 µm. Ctrl: control, standard diet; EtOH: 8 or 12 weeks of ethanol in the standard diet; EtOH + Probiotic: 8 or 12 weeks of ethanol plus *L. fermentus LF31* in the standard diet.

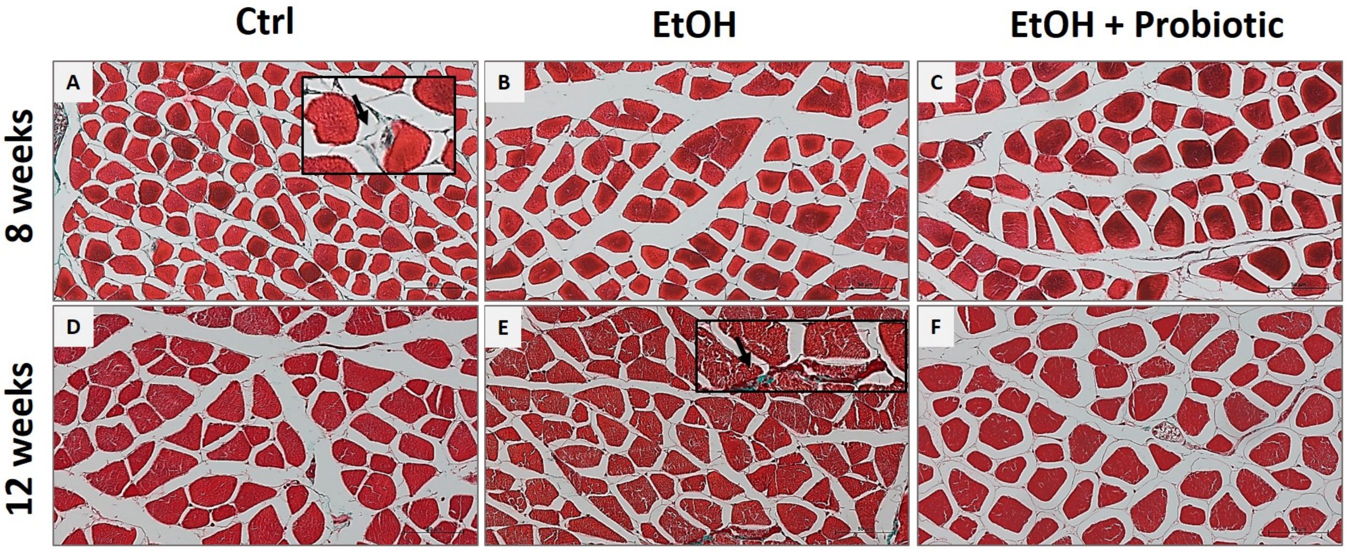

Figure S2. Masson's trichrome stain of the *red gastrocnemius* muscle. Representative images show muscle fibers with polygonal morphology, characterized by a deep red cytoplasm, indicative of myofiber staining. Connective tissue (in green) is visible in the endomisial and perimysial spaces, separating the muscle fibers. In the two time points and the three experimental groups, no particular signs of fibrosis are observed, other than those physiologically present within the muscle tissue. Magnification 200X. Scale bar 50 μm. Ctrl: control, standard diet; EtOH: 8 or 12 weeks of ethanol in the standard diet; EtOH + Probiotic: 8 or 12 weeks of ethanol plus *L. fermentum LF31* in the standard diet.
